# Response to letter to the editor

**DOI:** 10.5620/eaht.2023022

**Published:** 2023-10-27

**Authors:** Onyenekenwa Cyprian Eneh

Response to letter to the editor

On behalf of O.C. Eneh et al., the authors of “Mitigating potential public health risks and challenges from hazardous materials contained in electronic waste items in a developing country setting”, Environmental Analysis Health and Toxicology, 38(1). DOI: https://doi.org/10.5620/eaht.2023001, I write in response to a rejoinder titled Letter to editor A Pesticide Decision Support Tool to guide the selection of less environmentally harmful pesticides for the sugar cane industry by Slamet Wardoyo of the Department of Environmental health, Poltekkes Kemenkes Surabaya, Surabaya, Indonesia.

The rejoinder applauded the author's efforts to raise awareness and propose solutions for achieving the Sustainable Development Goals (SDGs) targets related to hazardous chemicals and waste management, but raised some concerns and recommendations, such as (a) acknowledging the benefits and opportunities of e-waste recycling and reuse, (b) provision of additional data and examples to support the claims and recommendations, and (c) the use of language and a format that is more reader-friendly. The rejoinder recommended that authors revise and update their article to be more objective, specific, evidence-based, interesting, and approachable for the readers.

Congratulations to the EAHT Editor on the wide, concerned and responsive readership. However, Slamet Wardoyo may wish to note that the objectives of the study were to (1) identify hazardous chemicals contained in significant quantities in ewaste items, and (2) describe their public health challenges and mitigation measures to enable stakeholders prepare basic information to serve as appropriate environmental health education technology policy (AEHETP) to guide the design of prophylactic, therapeutic and decontamination plans for awareness creation and raising to mitigate the toxic effects of ewaste items on users in a poor country setting.

Aside identifying hazardous chemicals contained in significant quantities in e-waste items, the article is concerned with public health challenges arising from exposure to the chemicals and how to mitigate them – not the positives and opportunities associated with the recycling and reuse of e-waste. Contrary to the submission of the rejoinder, the article contains sufficient data to address its objectives, claims and recommendations, including information to guide the formulation of appropriate environmental health education technology policy (AEHETP) for stakeholders use in designing education, preventive, therapeutic and decontamination plans. The paper needed not to explain what such a policy would entail or how it would be implemented and evaluated because this is outside the scope of the objectives of the paper, which is not a policy paper, nor is EAHT a policy journal. The paper is not a general audience article that ought to seek the general audience reader-friendliness, approval and appeal, hence its academic, technical and professional language and format. After all, before going to press, the article was checked by English and/or bibliographic experts for appropriate language and format.

Finally, it is sufficient for the paper to have achieved its objectives and made an important contribution to the body of research on hazardous chemicals and electronic waste in an academic, technical and professional language appropriate for the specific readership. Every other expectation of a reader could form the basis for future research, and not a reason or justification to re-write the paper.

